# Phylogeny and biogeography of the enigmatic ghost lineage Cylindrotomidae (Diptera, Nematocera)

**DOI:** 10.1038/s41598-021-91719-w

**Published:** 2021-07-06

**Authors:** Iwona Kania-Kłosok, André Nel, Jacek Szwedo, Wiktoria Jordan-Stasiło, Wiesław Krzemiński

**Affiliations:** 1 Department of Biotechnology, Institute of Biology and Biotechnology, University of Rzeszów, Zelwerowicza 4, 35-601 Rzeszów, Poland; 2 Institut Systématique Evolution Biodiversité (ISYEB), Muséum National d’Histoire Naturelle, CNRS, Sorbonne Université, Université des Antilles, EPHE, Paris, France; 3 Laboratory of Evolutionary Entomology and Museum of Amber Inclusions, Department of Invertebrate Zoology and Parasitology, University of Gdańsk, 59, Wita Stwosza St, 80308 Gdańsk, Poland; 4 Institute of Systematics and Evolution of Animals, Polish Academy of Sciences, Sławkowska 17, 31-016 Kraków, Poland

**Keywords:** Palaeontology, Evolution, Systems biology

## Abstract

Ghost lineages have always challenged the understanding of organism evolution. They participate in misinterpretations in phylogenetic, clade dating, biogeographic, and paleoecologic studies. They directly result from fossilization biases and organism biology. The Cylindrotomidae are a perfect example of an unexplained ghost lineage during the Mesozoic, as its sister family Tipulidae is already well diversified during the Cretaceous, while the oldest Cylindrotomidae are Paleogene representatives of the extant genus *Cylindrotoma* and of the enigmatic fossil genus *Cyttaromyia*. Here we clarify the phylogenetic position of *Cyttaromyia* in the stem group of the whole family, suggesting that the crown group of the Cylindrotomidae began to diversify during the Cenozoic, unlike their sister group Tipulidae. We make a comparative analysis of all species in *Cyttaromyia*, together with the descriptions of the two new species, *C. gelhausi* sp. nov. and *C. freiwaldi* sp. nov., and the revision of *C. obdurescens*. The cylindrotomid biogeography seems to be incongruent with the phylogenetic analysis, the apparently most derived subfamily Stibadocerinae having apparently a ‘Gondwanan’ distribution, with some genera only known from Australia or Chile, while the most inclusive Cylindrotominae are Holarctic.

## Introduction

Cylindrotomidae Schinner, 1863^1^ together with Limoniidae Speiser, 1909^2^, Pediciidae Osten-Sacken, 1860^3^, and Tipulidae Latreille, 1802^4^ sensu stricto are classified within Tipuloidea Latreille, 1802^4^, group of insects present in the fossil record since at least 220 Ma (Triassic)^5^. With only 71 extant^6^ and 16 extinct species^7^, this smallest family within Tipuloidea is divided into two subfamilies Cylindrotominae, represented mainly in Holarctic Region, and Stibadocerinae with an example of vicariant distribution with a sister-group relationship between South American and East Asian taxa, supporting hypothesis an ‘ancestral’ trans-Pacific biota^8^.

According to phylogenetic synthesis based on combined morphological characters of adult, larvae and pupae, together with nuclear gene sequence data as 28S rDNA or CAD, the Cylindrotomidae are found as a sister group of Tipulidae (both being treated as subfamilies in Tipulidae in Ref.^9^. This group of insects is generally indicated as a sister group or being closely related to the Tipulidae by other authors^10–20^. But, within Cylindrotomidae^21^, only the representatives of subfamily Cylindrotominae are known from fossil record. The oldest described representatives of Cylindrotominae are only known from the Paleogene (56.0–47.8 Ma) by the extant genera *Cylindrotoma* Macquart, 1834^22^ and *Diogma* Edwards, 1938^23–33^, plus the extinct genus *Cyttaromyia* Scudder, 1877^7,34^, while the oldest stem Tipulidae are Jurassic and the oldest crown Tipulidae are Cretaceous^5,35–37^. Thus, the Cylindrotomidae can be considered as a typical ghost lineage during the Mesozoic.

This phytophagous group of craneflies, which immatures lives among mosses and herbaceous plants.

The Cylindrotomidae (Supplementary Data S1) probably knew a period of diversification during the Eocene, sufficient to become frequent enough to be found as fossils. Most of the fossil Cylindrotomidae are know from the Middle Eocene Baltic amber^31,32,38,39^ and the Late Eocene Florissant Formation in USA^24,39^. Three species of *Cylindrotoma* are known from impressions of the Ypresian Fur Formation; two species from the Ølst Formation of Denmark were described within *Cyttaromyia*^29^ (Supplementary Table S1). Four species of *Cyttaromyia* were described from the Eocene Green River Formation USA, the other were described from Florissant Formation and Kishenehn Formation in USA, Middle Salt Formation in Alsace (France), Biamo Formation in Russia, and from Baltic amber^24,27,28,30,31,40^. Here we propose a morphological phylogenetic analysis to define the relationships between extinct genus *Cyttaromyia* and the other taxa in the family. We also describe two new species of *Cyttaromyia* on the basis of new fossils from the same Formation, and new technics of research give us possibility to redescribe *Cyttaromyia obdurescens* Cockerell, 1924^27^.

## Results

### Systematic paleontology

Order Diptera Linnaeus, 1758^42^

Infraorder Tipulomorpha Latreille, 1802^4^

Family Cylindrotomidae Schinner, 1863^1^

Subfamily Cylindrotominae Schinner, 1863^1^

Genus *Cyttaromyia*

Type species: *Cyttaromyia fenestrate* Scudder, 1877^34^, by monotypy.

Key to species of the genus *Cyttaromyia* Scudder, 1877^34^

1. Wings without distinct patterning………………..………………………….…...…...………………………3.
  Distinct patterns of coloration on wings………………..………………………….…...…...…………2.
2. Rs longer than R_2+3+4_ and R_3+4_ combined………………..………………………….…...…...***Cyttaromyia vahldieki*** Freiwald, 1991^29^
                                                                                                                                                                                                                                                               Denmark/Ølst Formation
  Rs shorter than R_2+3+4_ and R_3+4_ combined………………..………………………….…...…...***Cyttaromyia rayona*** Freiwald & Krzemiński, 1991^30^
                                                                                                                                                                                                                                                                Russia/Biamo Formation

3. Wings hyaline………………..………………………….…...…...…………………………………………………………4.
  Wings pale brownish without conspicuous markings with end of marginal cell apically somewhat clouded (Cockerell, 1924)………………..………………………….…...…...***Cyttaromyia reclusa*** Cockerell, 1924^26^
                                                                                                                                                                                                                      USA/Green River Formation/Roan Moutains/Colorado

4. Vein R_1_ well-developed………………..………………………….…...…...………………………………5.
  Vein R_1_ reduced………………..………………………….…...…...……………………………………7.
5. Crossvein m-cu situated beyond bifurcation of Mb on M_1+2_ and M_3+4_; Sc terminating in C far beyond fork of Rs………………..………………………….…...…...……………………………………………………6.
  Crossvein m-cu situated at bifurcation of Mb on M_1+2_ and M_3+4_; Sc terminating in C just beyond fork of Rs………………..………………………….…...…...***Cyttaromyia gelhausi*** **sp. nov.**
                                                                                                                                                                                                                                                    USA/Green River Formation

6. Crossvein sc-r one of its length before tip of Sc; A_1_ tip before tip of Sc level………………..………………………….…...…...***Cyttaromyia obdurescens*** Cockerell, 1925^27^
                                                                                                                                                                                                                     USA/Green River Formation/Roan Moutains/Colorado
  Crossvein sc-r at least two of its length before tip of Sc; A_1_ tip beyond e tip of Sc level………………..………………………….…...…...***Cyttaromyia princetoniana*** Scudder, 1894^24^
                                                                                                                                                                                                                                                             USA/Green River Formation

7. Sc elongate, terminating in C well beyond level of fork of Rs………………..………………………….…...…...8.
  Sc short, terminating in C before level of fork of Rs………………..………………………….…...…...……………***Cyttaromyia freiwaldi*** sp. nov.
                                                                                                                                                                                                                                                      USA/Green River Formation

8. Crossvein m-cu situated at most or before fork of Mb on M_1+2_ and M_3+4_…9.
  Crossvein m-cu situated beyond fork of Mb on M_1+2_ and M_3+4_………………..………………………….…...…...***Cyttaromyia frelloi*** Krzemiński, 1998^31^
                                                                                                                                                                                                                                                                                     Baltic amber

9. Vein r–r (R_2_) terminating before level of r′–m′, before level of m–m………………..………………………….10.
  Vein r–r (R_2_) terminating at level of r′–m′, behind level of m–m………………..………………………….…...…...***Cyttaromyia fenestrata*** Scudder, 1877^34^
                                                                                                                                                                                                                                                      USA/Green River Formation

10. d′-cell as long as d-cell or shorter………………..………………………….…...…...……………11.
  d′-cell longer tan d-cell………………..………………………….…...…...…………………………………12.
11. M_3+4_ bifurcation on M_3_ and M_4_ approximately at level of m-m; d-cell 2× as long as M_4_; m-m beyond level of tip of r–r (R_2_)………………..………………………….…...…...***Cyttaromyia lynnae*** De Jong, 2019^40^
                                                                                                                                                                                                                                                             USA/Kishenehn Formation.
  M_3+4_ bifurcation on M_3_ and M_4_ before level of m–m; d-cell 1.5× as long as M_4_; m–m approximately at level of tip of r–r (R_2_)………………..………………………….…...…...***Cyttaromyia fuscula*** Cockerell, 1921^25^.
                                                                                                                                                                                                                                                    USA/Green River Formation

12. Fork of Mb at level of fork of Rs; d′-cell narrowed at base………………..………………………….…...…...***Cyttaromyia quievreuxi*** Séguy, 1934^28^
                                                                                                                                                                                                                                            Alsace, France/Middle Salt Formation.
  Fork of Mb before level of fork of Rs; d′-cell narrow, but not narrowed at base………………..………………………….…..13.

13.                                                                                                                                                                                                                                           Tip of r–r (R_2_) beyond level of tip of fork of M_3 +4_ on M_3_ and M_4_; Rs at 2× as long as R_2+3+4_………………..………………………….…...…...***Cyttaromyia scudderi*** Freiwald, 1991^29^
                                                                                                                                                                                                                                                        Denmark/Ølst Formation
  Tip of r–r (R_2_) before level of tip of fork of M_3 +4_ on M_3_ and M_4_; Rs at approximately as long as R_2+3+4_………………..………………………….…...…...***Cyttaromyia rossi*** Krzemiński, 2019^41^
                                                                                                                                                                                                                                          UK/Isle of Wight/Bembridge Marls

***Cyttaromyia obdurescens*** Cockerell, 1925^27^

(Fig. 1).

**Figure 1:**
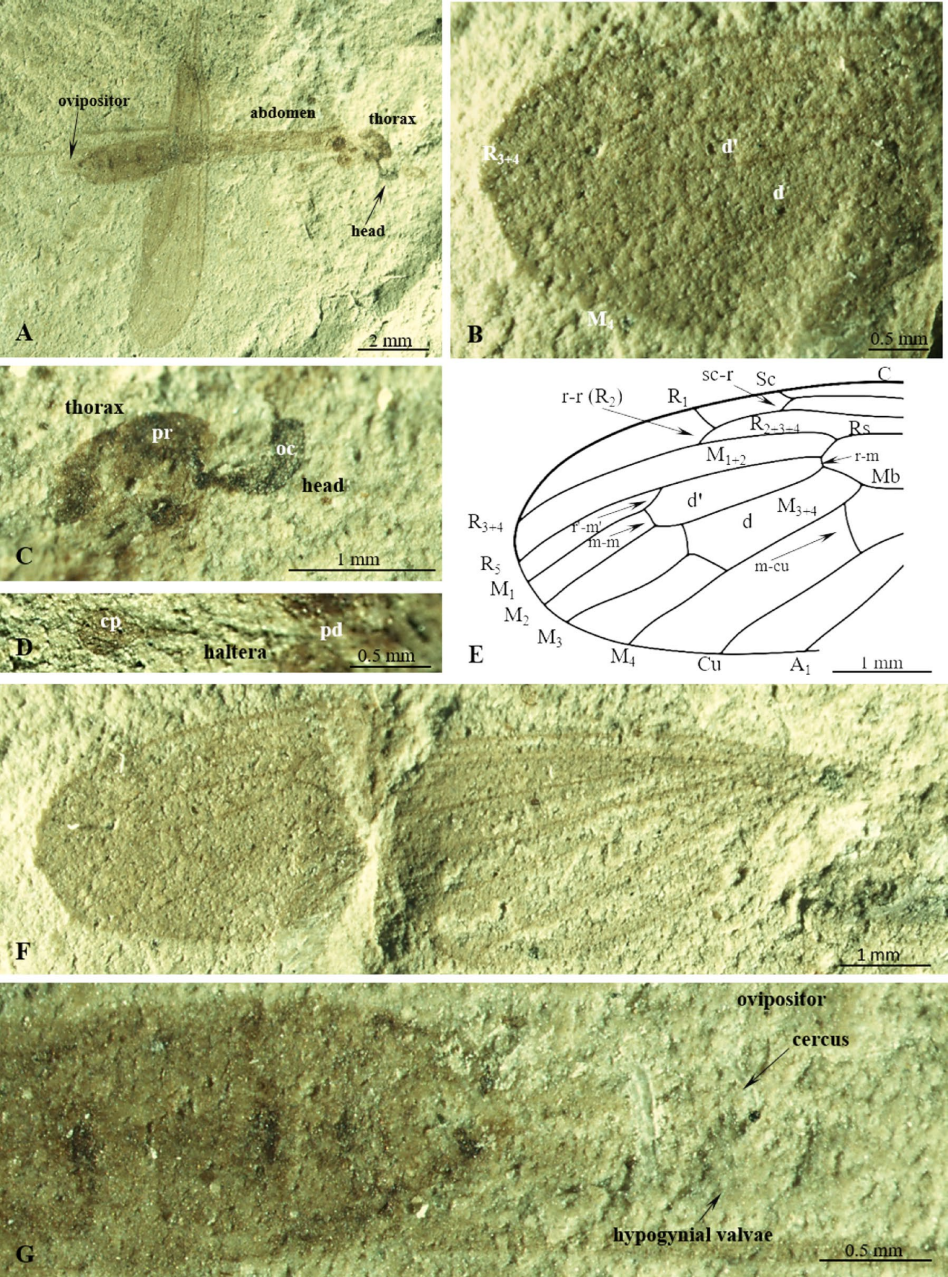
*Cyttaromyia obdurescens* Cockerell, 1925^27^*,* holotype No. 26284 (AMNH) (female): (**A**) habitus, lateral view; (**B**) apex of wing; (**C**) head and thorax, enlarged lateral view; (**D**) haltera; (**E**) apex of wing, drawing; (**F**) wing; C. terminal part of abdomen with *ovipositor* visible. cp, *capitellum*, pd, *pedicellus* of haltera, oc, *ocellus*, ped, *pedicellus*, pr, *pronotum*.

#### Material examined

Holotype No. 26284 (AMNH) (female); American Museum National History; Green River Formation USA, Eocene.

#### Emended diagnosis

Wing without color spots; Sc elongate, terminating in C well beyond level of fork of Rs, beyond r–m level but far before m–m and r′–m′ level; opposite approximately half the length of R_2+3+4_; vein r–r (R_2_) terminating far before r′–m′ and m–m level, at level of basal part of M_3_; R_1_ well-developed; R_2+3+4_ longer than half length of Rs; d′-cell longer than d-cell, narrowed at its base; crossvein m-cu positioned beyond fork of Mb on M_1+2_ and M_3+4_; apical section of M_3_ almost as long as d-cell; A_1_ tip positioned near apex of wing, far behind level of Mb bifurcation on M_1+2_ and M_3+4_, before r–m level.

#### Comparison

*Cyttaromyia obdurescens* differs from *C. fenestrata*, *C. freiwaldi* sp. nov., *C. frelloi*, *C. fuscula*, *C. lynae*, *C. quievreuxi*, *C. scudderi*, and *C. vahldieki* by a well-developed vein r–r (R_2_). Wing of *C. obdurescens* is without spot. *C. rayona* and *C. vahldieki* have different patterning of wings. In contrast to *C. gelhausi* sp. nov., crossvein m-cu is situated beyond Mb, while in *C. gelhausi* sp. nov., it is located at Mb bifurcation, in *C. frelloi* just before Mb bifurcation. In *C. obdurescens*, vein sc-r is located one of its length from tip of Sc, tip of A_1_ is located before tip of Sc, while in *C. princetoniana* vein sc-r is located at least two of its length before the tip of Sc, A_1_ tip is located beyond level of tip of Sc. In *C. obdurescens* crossvein m-cu is positioned beyond fork of Mb measured from base of wing, while in *C. rossi*, m-cu is distinctly before fork of Mb.

### *Cyttaromyia gelhausi* sp. nov.

http://zoobank.org/urn: lsid:zoobank.org:act:9165E3D2-514B-4524-9F85-A9895CBF2A31.

(Figs. 2, 3).

**Figure 2:**
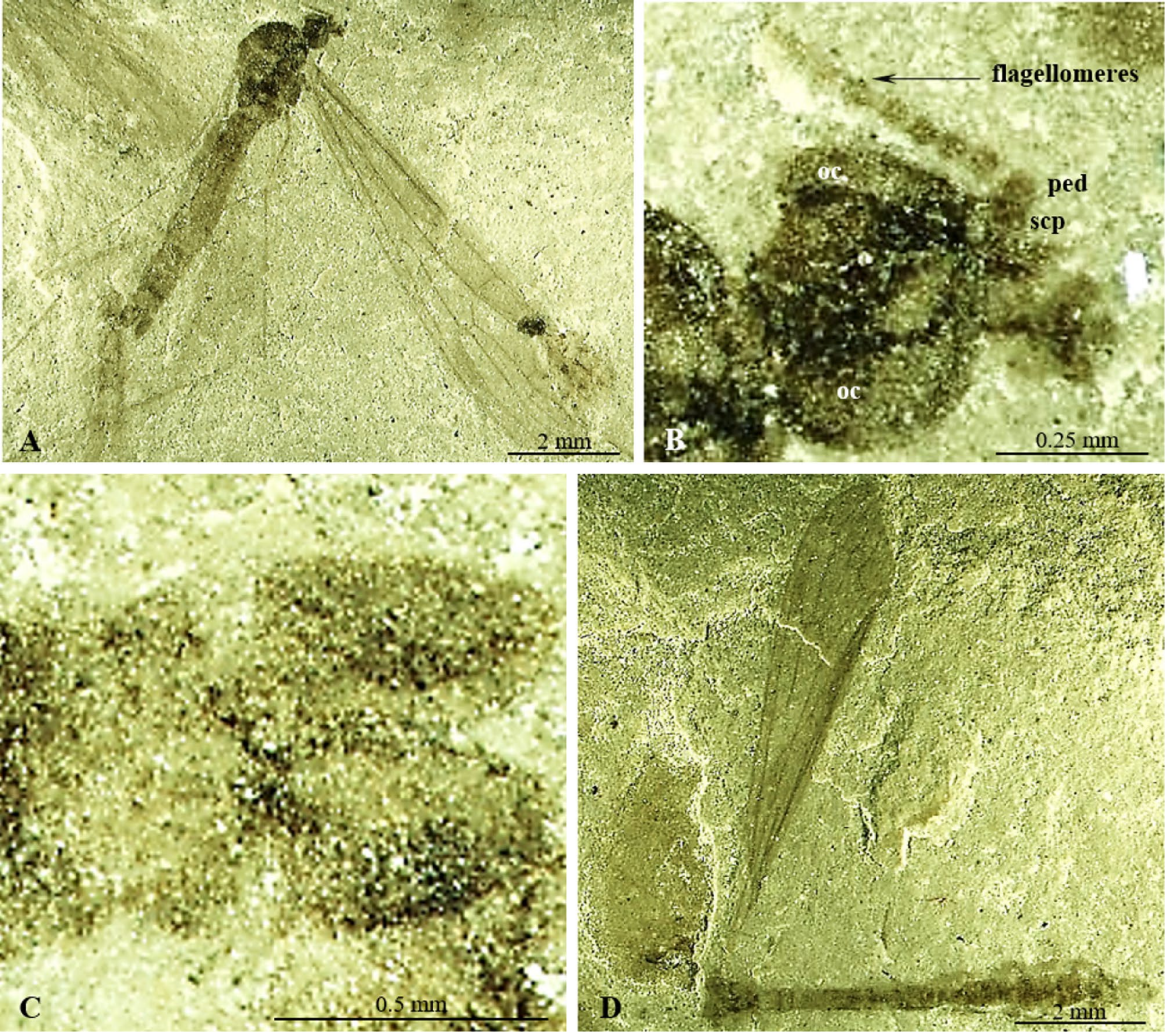
*Cyttaromyia gelhausi* sp. nov.: A.-C. No. MNHN.F.A71341 (18a, male) (holotype): (**A**) habitus, latero-ventral view; (**B**) head, ventral view; (**C**) hypopygium, ventral view; D. No. MNHN.F.A71341 (18c, female) (additional material), habitus, lateral view. oc, *ocellus*; ped, *pedicellus*; scp, *scapus*.

**Figure 3:**
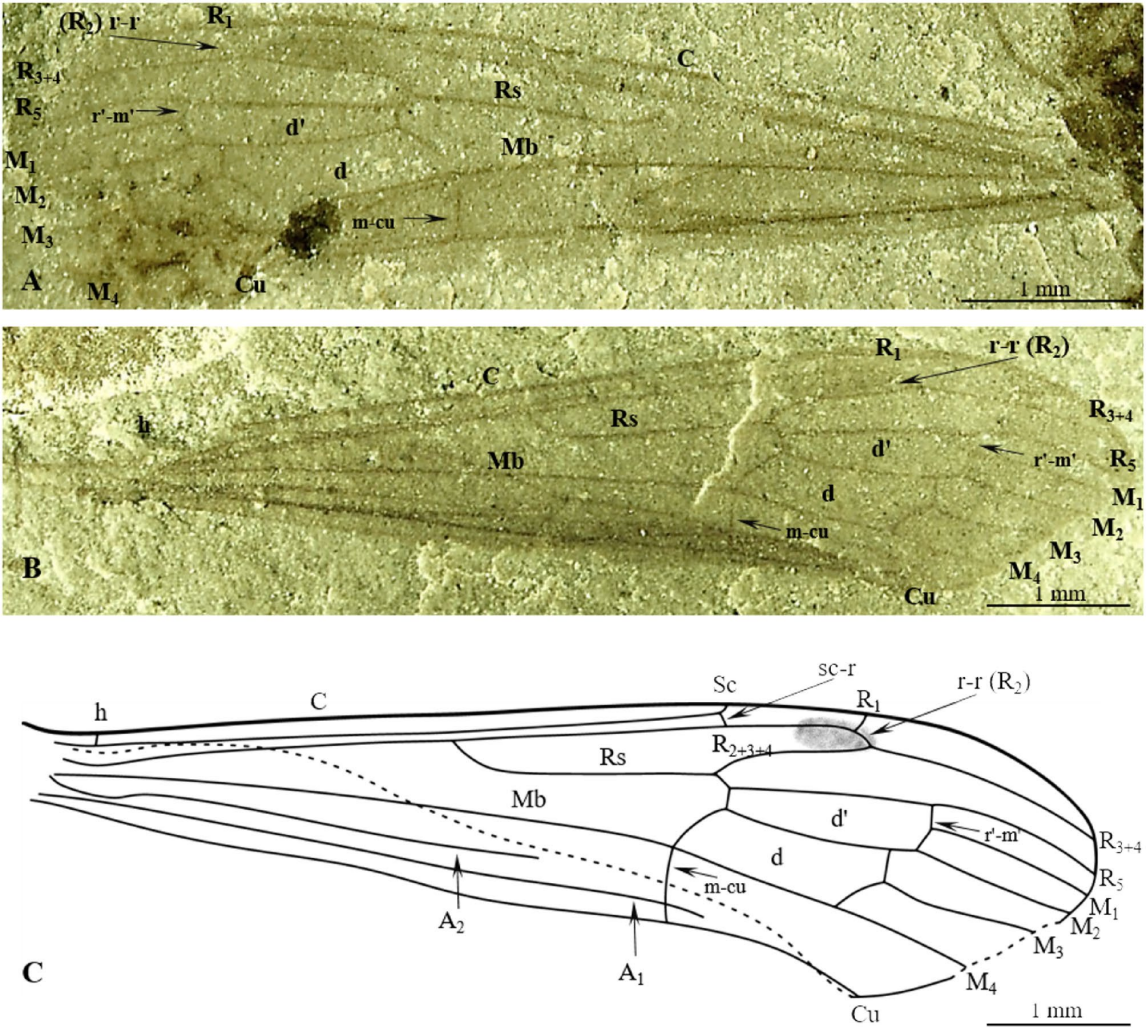
*Cyttaromyia gelhausi* sp. nov.: (**A**) No. MNHN.F.A71341 (18a, male) (holotype), wing; (**B**), C. No. MNHN.F.A71341 (18c, female) (additional material): (**B**) wing; (**C**) wing, drawing.

#### Material examined

Holotype MNHN.F.A71341 (18a, male); additional material 18b, female; 18c, female (on the same slab with holotype); Muséum national d'Histoire naturelle (MNHN), Paris; Green River Formation USA, Eocene.

#### Etymology

The specific name is given to honor Doctor John Gelhaus (Academy of Natural Sciences of Drexel University), the eminent specialist on extinct and extant insects.

#### Diagnosis

Flagellomeres short and relatively wide; wing without color spots; Sc not very elongate, terminating in C just beyond level of fork of Rs, opposite level of crossvein r–m; opposite approximately 1/10 length of R_2+3+4_; vein r–r (R_2_) terminating far before r′–m′ and just before m–m level, at level of basal part of M_3_; R_1_ well-developed; R_2+3+4_ longer than half length of Rs; d′-cell longer than d-cell, narrowed at its base; crossvein m-cu positioned at fork of Mb on M_1+2_ and M_3+4_; apical section of M_3_ almost as long as d-cell.

#### Comparison

*Cyttaromyia gelhausi* sp. nov. has no distinct color patterning of the wings in contrast to *C. vahldieki* and *C. rayona*. The wing of *C. reclusa* is pale brownish without conspicuous markings^26^, but the end of the marginal cell and the veins bounding the discal cell apically are somewhat clouded. The body length of *C. gelhausi* sp. nov. is at most 6.83 mm, wing length 7 mm, while the body length of *C. reclusa* is 13.5 mm and wing length 12 mm. In *C. gelhausi* sp. nov. tibial spurs are absent while pattern of tibial spurs of *C. frelloi* is 1:1:2^31^. Vein R_1_ is well-developed in *C. gelhausi* sp. nov., while in *C. freiwaldi* sp. nov., *C. frelloi*, *C. fuscula*, *C. lynnae*, *C. quievreuxi*, *C. rossi*, *C. scudderi*, *C. vahldieki* R_1_ is reduced. In *C. gelhausi* sp. nov. Sc is not very elongate, terminating in C just beyond level of fork of Rs, opposite the level of crossvein r-m, while in *C. fenestrata*, *C. frelloi*, *C. fuscula*, *C. lynnae*, *C. obdurescens*, *C. princetoniana*, *C. quievreuxi*, *C. rayona*, *C. scudderi*, and C. *vahldieki*, it is terminating far beyond level of Rs. In *C. freiwaldi* sp. nov. Sc terminating in C before Rs level. In *C. fenestrata* and *C. vahldieki*, Sc is very elongated, terminating in C opposite basal part of M_3_. Crossvein m-cu is positioned at fork of Mb on M_1+2_ and M_3+4_ while in other fossil species of the genus *Cyttaromyia* this vein is situated beyond fork of Mb; in *C. frelloi* this vein is located before fork of Mb. Moreover, in *C. vahldieki* and *C. fenestrata* vein r–r (R_2_) terminating in R_3+4_ beyond d′ level, far beyond the level of basal part of M_3_, in C. *gelhausi* sp. nov. this vein is terminating at the level of d’, at the level of basal part of M_3_.

### *Cyttaromyia freiwaldi* sp. nov.

(Figs. 4, 5).

**Figure 4:**
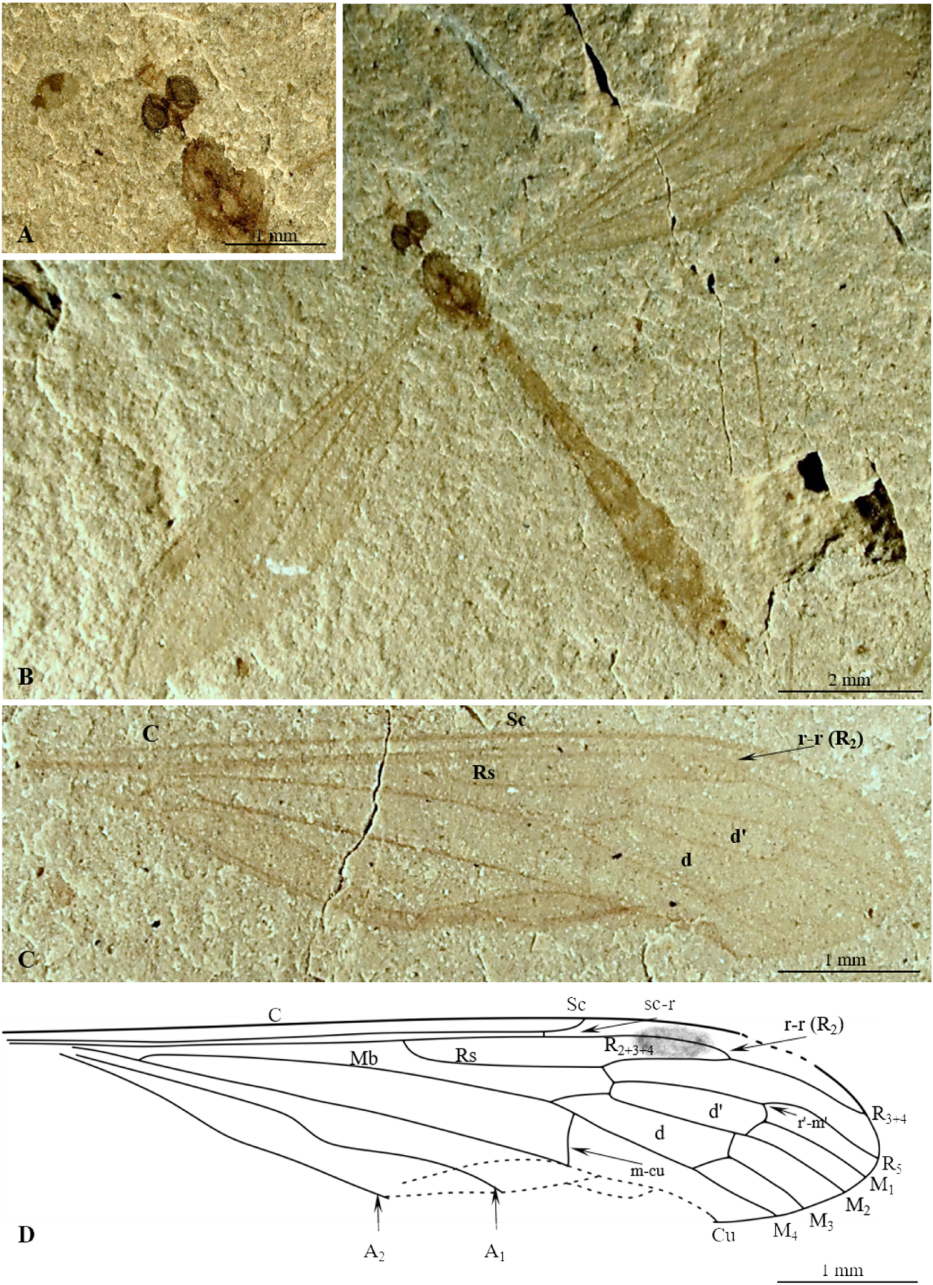
*Cyttaromyia freiwaldi* sp. nov. No. MNHN.F.A71342 (70A, part, female), (holotype): (**A**) habitus, dorsal view; (**B**) head, dorsal view; (**C**) ovipositor, dorsal view, (**D**) wing venation, (**E**) wing venation, drawing.

**Figure 5:**
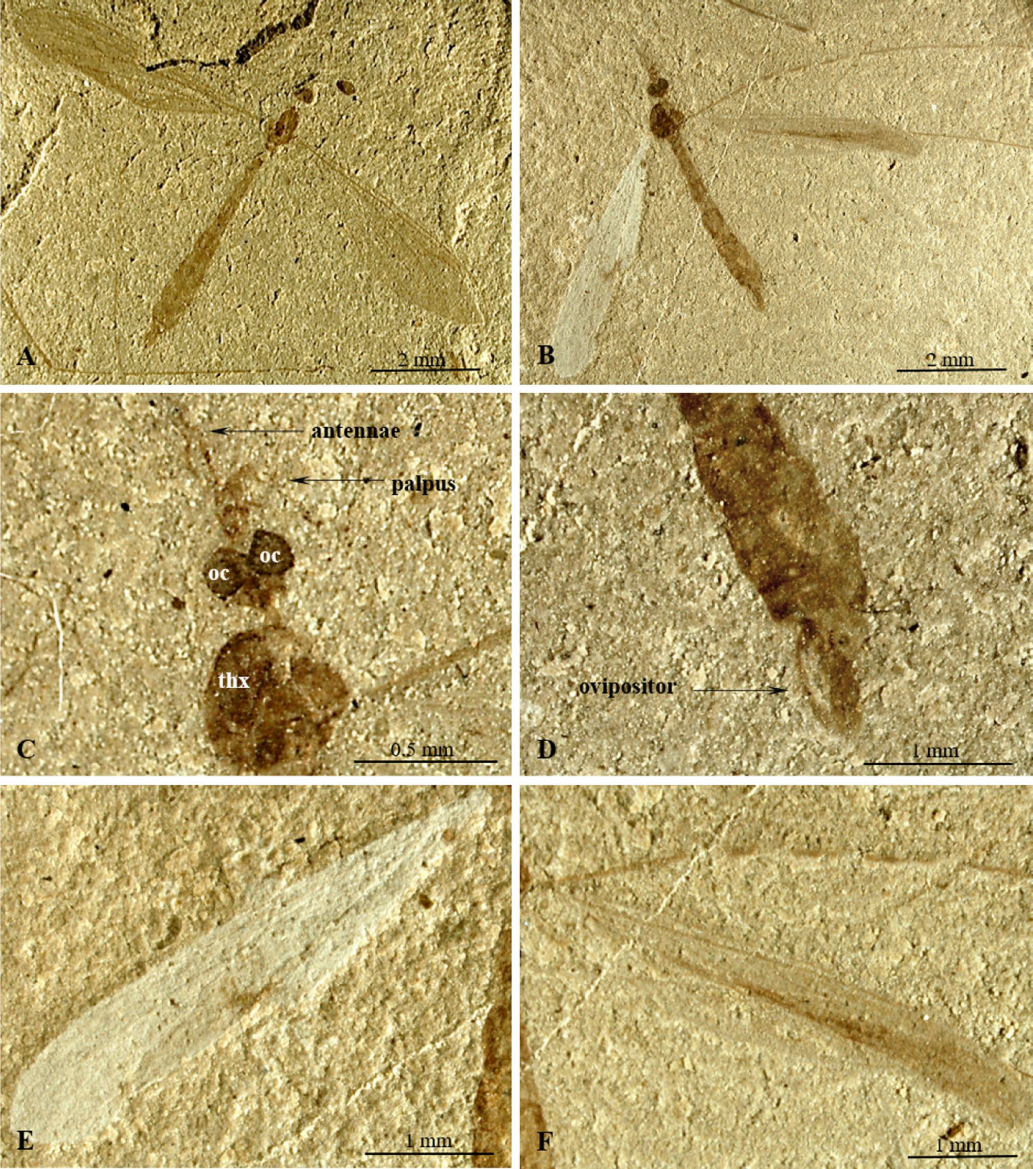
*Cyttaromyia freiwaldi* sp. nov.: (**A**) No. MNHN.F.A71342 (68, counterpart, female), (holotype), habitus, dorsal view; B.-F. No. MNHN.F.A71342 (70AB, female), (additional material): (**B**) habitus, dorsal view; (**C**) head, dorsal view; (**D**) terminal part of abdomen with ovipositor visible, dorsal view; (**E**) left wing; (**F**) right wing. oc, ocelli; thx, thorax.

http://zoobank.org/urn: lsid:zoobank.org:act:DB604450-AD47-4644-82E0-A33FEAEB7157.

#### Material examined

Holotype MNHN.F.A71342 (70A, part/68, counterpart, female), additional material 70B, female, on the same slab as holotype), Muséum national d'Histoire naturelle (MNHN), Paris; Green River Formation USA, Eocene.

#### Etymology

The new species is dedicated to the German eminent researcher Doctor Andre Freiwald (Institut für Paläontologie, Universität Erlangen).

#### Diagnosis

Wing without color spots; Sc short, terminating in C before level of fork of Rs, far before level of crossvein r–m; vein r–r (R_2_) terminating far before r′–m′ level and at m-m level, just beyond level of basal part of M_3_; R_1_ atrophied; R_2+3+4_ longer than half length of Rs; d′-cell shorter than d-cell, narrowed at its base; crossvein m-cu positioned beyond fork of Mb on M_1+2_ and M_3+4_; M_3_ shorter than d-cell.

#### Comparison

*Cyttaromyia freiwaldi* sp. nov. has no distinct patterning of the wings in contrast to *C. vahldieki* and *C. rayona*. In contrast to *C. reclusa*, the body length of *C. freiwaldi* sp. nov. is at most 4.8 mm, wing length 6.22 mm, while the body length of *C. reclusa* is 13.5 mm and wing is 12 mm long with the end of the marginal cell and the veins bounding the discal cell apically are somewhat clouded^26^. *C. freiwaldi* sp. nov. differs from other fossil species especially by point of termination of Sc. In *C. freiwaldi* sp. nov. vein Sc terminating in C before fork of Rs while in other fossil species, excluding *C. reclusa*^27^, Sc terminating in C just beyond or far beyond bifurcation of Rs. Moreover, in contrast to *C. gelhausi* sp. nov., *C. obdurescens*, *C. princetoniana*, and *C. rayona*, the vein R_1_ in *C. freiwaldi* sp. nov. is reduced. Vein r–r (R_2_) of *C. freiwaldi* is terminating in R_3+4_ at d-cell level while in *C. fenestrata* and *C. rayona* beyond this level, in *C. vahldieki* even beyond d`-cell level (Supplementary Data S2).

### Phylogenetic position of *Cyttaromyia* within Cylindrotomidae

**Figure 6:**
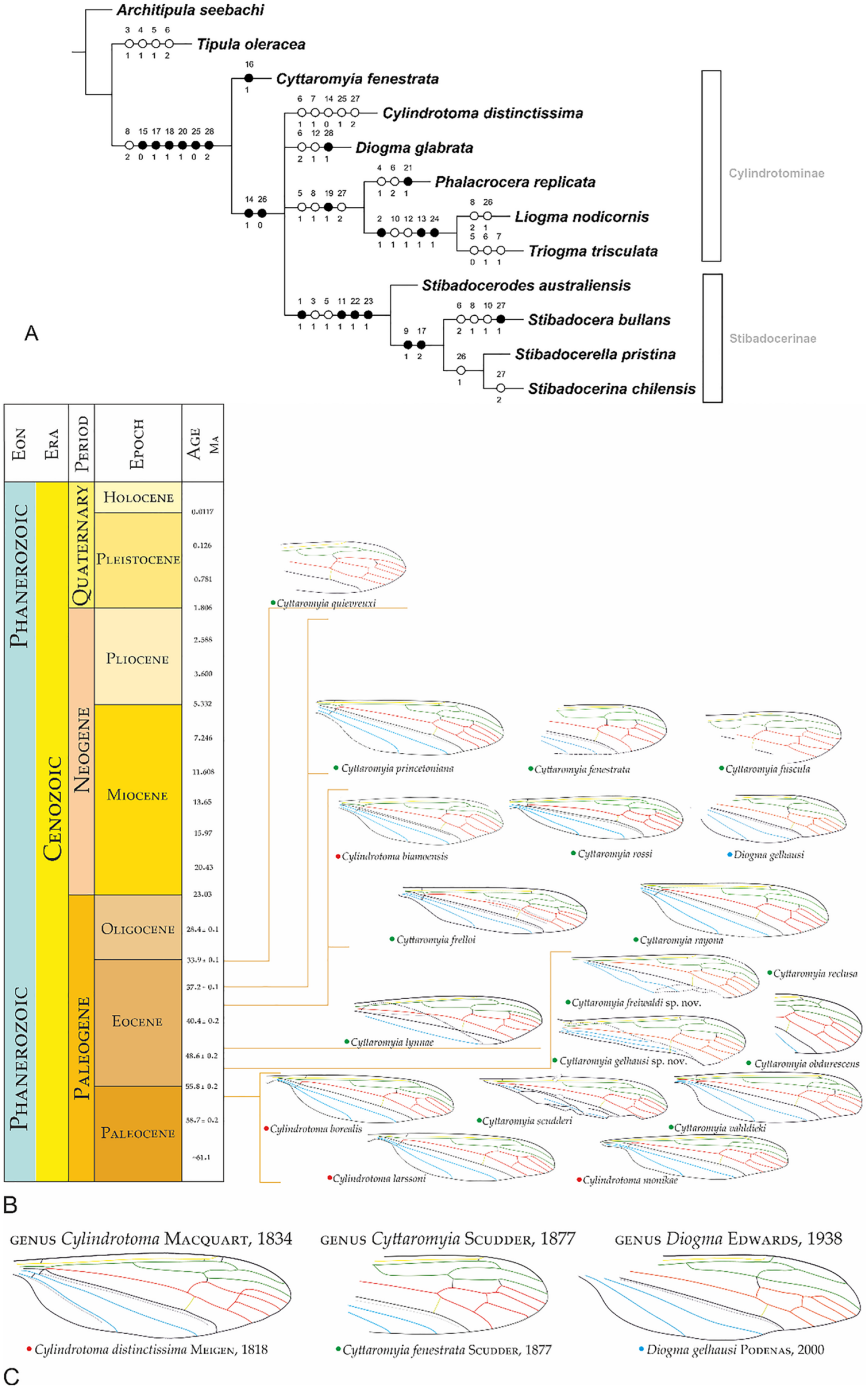
(**A**) Consensus relationships tree of genera of subfamily Cylindrotominae. Filled circles indicate synapomorphies or autapomorphies; open circles indicate plesiomorphies. Number of character given above circles, states of characters below circles; (**B**) Wing venation of fossil Cylindrotominae with chronostratigraphic distribution view; (**C**) Wing venations of representatives of genera: *Cylindrotoma*, *Cyttaromyia*, *Diogma*, represented in fossil record. Wing venation redrawing^24,25,27–29,31,34,42,50^. Stratigraphic chart according to International Stratigraphic Chart, International Commission of Stratigraphy (v. 2021/05) https://stratigraphy.org/chart.

The parsimony analysis yielded three equally most parsimonious cladograms, 53 steps long, with consistency index CI = 62, RI = 66. Their consensus majority rule cladogram is shown in Fig. 6A. The Cylindrotomidae clade is supported by six synapomorphies: presence of petiole (character 15, state 0), relationship of R_3_ and R_4_ (character 17, state 1), position of crossvein m-cu relative to the bifurcation of M_3+4_ (character 18, state 1), shape of d-cell (character 20, state 1), position of tip of A_2_ (character 25, state 0), morphology of aedeagus (character 28, state 2). *Cyttaromyia* is supported on consensus tree by the ‘presence of supernumerary crossvein connecting vein R_4+5_ with M_1_ near its origin, to produce two discal cells’ (character 16, state 1). The clade (Cylindrotominae + Stibadocerinae (Hennig, 1973)^43^) is supported by two synapomorphies, relationship of M_1_ and M_2_ (character 14, state 1), position of tip of A_2_ (character 26, state 0). The clade [*Phalacrocera replicata* Linnaeus, 1758^41^ + (*Liogma nodicornis* Osten Sacken, 1865^44^ + *Triogma trisculata* Shummel, 1829^45^)] is supported by one synapomorphy, viz. position of crossvein m-cu relative to the bifurcation of Mb (character 19, state 1). *Triogma trisculata* appears as the sister-group to *Liogma nodicornis*, the clade (*Liogma nodicornis* + *Triogma trisculata*) being supported by three synapomorphies: shape of flagellomeres (character 2, state 1), position of R_5_ (character 13, state 1), degree of reduction of crossvein r–m (character 24, state 1). The clade Stibadocerinae [= (*Stibadocerodes australiensis* Alexander, 1922^46^ + (*Stibadocera bullans* Enderlein, 1912^47^) + (*Stibadocerella pristina* Brunetti, 1918^48^ + *Stibadocerina chilensis* Alexander, 1928^49^)))] is supported by two synapomorphies: the number of branches of Rs reaching wing margin (character 9, state 1), relationship of R_3_ and R_4_ (character 17, state 2) (Supplementary Data S3).

## Discussion

The subfamily Cylindrotominae^21^ currently contains more species and genera, compared to the Stibadocerinae. The oldest record of the extinct genus *Cyttaromyia* is Paleogene, as for the genus *Cylindrotoma*. The fossil record of the Cylindrotominae dated back to at least 56.0 Ma^29^, with no evidences on older occurrences. Other representatives of Cylindrotominae are known in the Eocene^24,25,27,28,31,32,38^, but most of them are strictly modern^6^ (Supplementary Figs. S1, S2).

According to our parsimony analysis (consensus tree), *Cyttaromyia* falls as sister group of all the extant genera of Cylindrotomidae, and thus belongs to the stem group of the family, and could correspond to a different subfamily. Also, the extant Cylindrotominae appear paraphyletic in respect to the Stibadocerinae because the two genera *Cylindrotoma* and *Diogma* fall in an unresolved polytomy with this subfamily plus a clade that contains the other cylindrotomine genera. Nevertheless, these results are preliminary and would need to be completed by the addition of characters, in particular molecular.

The Cylindrotominae (and also *Cyttaromyia*) have a Holarctic distribution, while the Stibadocerinae have a more disjunctive distribution in Indo-Malaysia, Australo-Papua and Southern Neotropics (Taiwan, China, Indonesia, Malaysia, India, Papua New Guinea, Philippines, Australia, and Chile). Such distribution resembles that of an ancient Gondwana group, with ‘relic’ taxa in Australia and Chile; but the present phylogenetic analysis would contradict this hypothesis, as the only known stem representative of the family is also Holarctic. Further analyses together with discoveries of fossil Cylindrotomidae in the Southern Hemisphere shall be necessary to clarify this complex, strange situation.

From a taxonomic point of view, *Architipula* is characterized by the occurrence of vein Sc tip beyond fork of Rs level, subequal to or a little shorter than veins R_2+3_ and R_3_ combined, distinctly inclined crossvein m-m between M_1+2_ and M_3_, usually short and straight vein A_2_^51^ *Cyttaromyia* is characterized by the occurrence of two discal cells (d-cell and d′-cell), supernumerary crossvein r′–m′ connecting vein R_5_ with M_1_ near its origin, to produce two discal cells. Some similarities are present in the wing venations of *Cyttaromyia* and *Cylindrotoma*: separate M_1_ and M_2_ and relatively long vein M_1_*.* In *Cylindrotoma* the crossvein r′–m′ is atrophied, but the base of vein M_1_ is strongly arched and only one discal cell (d-cell) is present. In all other Cylindrotominae, like *Diogma* (recorded from the Middle Eocene)^32^, or other genera with a younger fossil record, the crossvein r′–m′ is reduced and only one discal cell (d-cell) is present (Fig. 6B,C; Supplementary Figs. S3, S4).

## Conclusion

The revision of *Cyttaromyia obdurescens* and the description of two new species *Cyttaromyia gelhausi* sp. nov. and *Cyttaromyia freiwaldi* sp. nov., allowed us to propose a key to the species of this genus. We have also made the first morphological phylogenetic analysis of the Cylindrotomidae, with in the rather surprising result of the putative paraphyly of the Cylindrotominae and a position of *Cyttaromyia* in the stem group of this family.

## Material and methods

The study was based on material from the collection of the Muséum national d'Histoire naturelle (MNHN), Paris (five specimens) and American Museum National History (AMNH) (one specimen). The imprints from sediments of Green River Formation USA (age 50.3–46.2 Ma^42^) were studied using a Nikon SMZ 1500 stereomicroscope equipped with a Nikon DS–Fi1 camera in University of Rzeszów. The microphotographs and measurements were taken with NIS–Elements D 3.0 software. Drawings were completed by tracing the photographs, nomenclature of wing venation was used^31^.

The Eocene Green River Formation USA (50.3–46.2 Ma) (Rocky Mountains, Colorado, Wyoming, Utah) is one of the most famous Eocene palaeontological sites of the World. The sediments include mainly calcium carbonate, calcite and aragonite. The occurrence of different types of sludge varies with the geological levels. Tipton Shale Member in Greater Green River Basin is the oldest rock formation of the Green River^52^.

Placement of the genus *Cyttaromyia* within Cylindrotomidae was tested with the use of Maximum Parsimony (MP) criterion, implemented in TNT 1.5 software package, with the ‘Traditional Search’ options^53,54^, with memory to store 99,999 trees, 10,000 replications, with 100 trees to save per replication; utilizing tree-bisection-reconnection (TBR) algorithm and collapsing zero length branches. The type species of extinct and extant genera of family Cylindrotomidae were included in the analysis. *Architipula seebachi* (Geinitz, 1884)^55^—type species of the genus *Architipula* Handlirsch, 1906^56^, was selected as outgroup because the Architipulinae are closely related to Cylindrotominae. *Tipula oleracea* Linnaeus, 1758^41^ was used as a type species of the genus *Tipula*, in the family Tipulidae, currently considered as the sister family of the Cylindrotomidae. The morphological data to the matrix were compiled in the Nexus file using Mesquite v. 3.61 build 927^57^. All 28 characters of the imagines used in the analysis were treated as unordered and unweighted (Supplementary Table S2). Equal weighting analysis (EW) was performed^53^; the trees received were viewed and their features studied using WinClada 1.00.08 and ASADO 1.61, with Unambigous Changes Only, Fast Optimization (ACCTRAN) and Slow Optimization (DELTRAN) options^57–59^. Tree files received were adjusted using Corel Draw X3 and Photo-Paint Software. The 28 morphological characters of the imago observed in the fossil and recent material and used for analysis are listed below. The data matrix given is partly based on used morphological features^8,21,34,41,43–45,50,56^.

## Supplementary Information

Supplementary Information.

## References

[1] Schiner JR (1863). Vorlaufiger Commentar zum dipterologischen Theile der Fauna austriaca. V [concl.]. Wiener Entomologische Monatschrift.

[2] Speiser P (1909). 4 Orthorapha. Orthorapha Nematocera. Wissenschaftliche ergebnisse der Schwedischen zoologischen expedition nach dem Kilimandjaro, dem Meru und den umgebenden Massaisteppen Deutsch-Ostafrikas 1905–1906, unter leitung von prof. dr. Yngve Sjostedt. Diptera.

[3] Osten Sacken CR (1860). New genera and species of North American Tipulidae with short palpi, with an attempt at a new classification of the tribe. Proc. Acad. Nat. Sci. Phila..

[4] Latreille, P. A. Histoire naturelle, generale et particuliere, des Crustaces et des Insectes. Tome troisieme. Ouvrage faisant suite a lhistoire naturelle generale et particuliere, composee par Leclerc de Buffon, et redigee par C.S. Sonnini, membre de plusieurs societes savantes. *Familles naturelles des genres, Paris*, 13–467. I–xii (1802).

[5] Krzemiński W (1992). Tipula (s. lato) *eva* n. sp. from Cretaceous (East Asia)—The oldest representative of the family Tipulidae (Diptera, Polyneura). Acta Zool. Crac..

[6] Oosterbroek, P. Catalogue of the Crane–flies of the World. (Diptera: Tipuloidea: Pedicidae, Limoniidae, Cylindrotomidae, Tipulidae). https://ccw.naturalis.nl/ Accessed 28 May 2020 (2020).

[7] Evenhuis, N. L. Catalog of the fossil flies of the world (Insecta: Diptera) website. Version. 2.0. http://hbs.bishopmuseum.org/fossilcat/ Accessed 13 Feb 2014 (2014).

[8] Ribeiro GC (2009). The Neotropical genus *Stibadocerina* Alexander and its phylogenetic relationship to other Stibadocerinae genera: Further evidence of an ancestral trans-Pacific biota (Diptera: Cylindrotomidae). Syst. Entomol..

[9] Petersen MJ, Bertone MA, Wiegann BM, Courtney GW (2010). Phylogenetic synthesis or morphological and molecular data reveals new insights into the higer level classification of Tipuloidea (Diptera). Syst. Entomol..

[10] Alexander CP (1919). Notes on the genus *Dicranoptycha* Osten Sacken (Tipulidae, Diptera). Entomol. News.

[11] Alexander CP (1920). New or little-known Tipulidae (Diptera). III. Ethiopian species. Ann. Mag. Nat. Hist..

[12] Savchenko EN (1966). Tipulidae. Fauna Ukrainy.

[13] Savchenko, E. N. Limoniidae of South Primorye. *Akademii Nauk Ukrainskoy SSR, Kiev* 1–156 (1983).

[14] Brodo, F. A revision of the genus *Prionocera* and the phylogeny of the family Tipulidae (Diptera). PhD Dissertation, *Carleton University, Ottawa, Ontario* (1984).

[15] Oosterbroek P, Theowald B (1991). Phylogeny of the Tipuloidea based on characters of larvae and pupae (Diptera, Nematocera), with an index to the literature except Tipulidae. Tijdschrift voor Entomologie.

[16] Starý J (1992). Phylogeny and classification of Tipulomorpha, with special emphasis on the famiy Limoniidae. Acta Zool. Crac..

[17] Ribeiro GC (2008). The phylogeny of the Limnophilinae (Limoniidae) and the early evolution of the Tipulomorpha (Diptera). Invertebr. Syst..

[18] Zhang X, Kang Z, Mao M, Li X, Cameron SL, de Jong H, Wang M, Yang D (2016). Comparative Mt genomics of the Tipuloidea (Diptera: Nematocera: Tipulomorpha) and its implications for the phylogeny of the Tipulomorpha. PLoS ONE.

[19] Lukashevich ED, Ribeiro GC (2019). Mesozoic fossils and the phylogeny of Tipulomorpha (Insecta: Diptera). J. Syst. Paleontol..

[20] Kang Z, Zhang X, Yang D (2019). Characterization of the complete mitochondrial genome of the snow crane-fly *Chionea crassipes gracilistyla* (Diptera, Tipuloidea, Limoniidae) with phylogenetic analysis. Mitochondrial DNA (B).

[21] Osten Sacken CR (1869). Monographs of the Diptera of North America. Part IV. Smithson. Misc. Collect..

[22] Macquart, P. J. M. Histoire naturelle des insectes. Diptères. Tome première. *N.E. Roret Paris*. 578 (1834).

[23] Edwards FW (2009). On the British Lestremiinae, with notes on exotic species—3. (Diptera, Cecidomyiidae). Proc. R. Entomol. Soc. Lond. (B).

[24] Scudder SH (1894). Tertiary Tipulidae, with special reference to those of Florissant, Colorado. Proc. Am. Philos. Soc..

[25] Cockerell TDA (1921). Eocene insects from the Rocky Mountains. Proc. U.S. Natl. Mus..

[26] Cockerell TDA (1924). Fossil insects in the United States National Museum. Proc. U. S. Natl. Mus..

[27] Cockerell TDA (1925). Plant and insect fossils from the Green River Eocene of Colorado. Proc. U. S. Natl. Mus..

[28] Séguy E (1934). Un nouveau Cylindrotomine fossile (Tipulidae). Encycl. Entomol. (B).

[29] Freiwald A (1991). Insekten aus der Fur-Formation von Dänemark (Moler, oberes Paleozän/ unteres Eozän). 5. Cylindrotomidae (Diptera: Tipulomorpha). Meyniana.

[30] Freiwald A, Krzemiński W (1991). Cylindrotomidae (Diptera, Tipulomorpha) from the Paleogene of Bolshaya Svetlovodnaya (eastern Asiatic USSR). Paläontol. Z..

[31] Krzemiński W (1998). *Cyttaromyia frelloi* sp. n., the first representative of the family Cylindrotomidae in Baltic amber (Diptera: Tipulomorpha). Polskie Pismo Entomologiczne.

[32] Podenas S (2000). A new species of *Diogma* Edwards, 1938 (Diptera, Cylindrotomidae) from Baltic amber (Eocene). Trans. Am. Entomol. Soc..

[33] Brodo F (1967). A review of the subfamily Cylindrotominae in North America (Diptera: Tipulidae). Univ. Kansas Sci. Bull..

[34] Scudder SH (1877). The first discovered traces of fossil insects in the American tertiaries. Bull. U. S. Geol. Geogr. Surv. Territ..

[35] Krzemiński W, Ansorge J (1995). New Upper Jurassic Diptera (Limoniidae, Eoptychopteridae) from the Solnhofen lithographic limestone (Bavaria, Germany). Stuttgarter Beiträge zur Naturkunde (B).

[36] Lukashevich ED (2009). Limoniidae (Diptera) in the Upper Jurassic of Shar Teg, Mongolia. Zoosymposia.

[37] Krzemiński W, Kopeć K, Kania I (2017). New and little known species from the genus *Leptotarsus* Guerin-Meneville, 1831 (Diptera: Tipulidae) from the Lower Cretaceous of Northern Brazil. Cretac. Res..

[38] Loew, H. Ueber den Bernstein und die Bernsteinfauna. *Programm der K. Realschule Meseritz* 1–44 (1850).

[39] Krzemiński W (1991). Revision of the fossil Cylindrotomidae (Diptera, Nematocera) from Florissant and White River, USA. Paläontol. Z..

[40] Krzemiński W, Blagoderov V, Azar D, Lukashevich E, Szadziewski R, Wedmann S, Nel A, Collomb F, Waller A, Nicholson DB (2019). True flies (Insecta: Diptera) from the late Eocene insect limestone (Bembridge Marls) of the Isle of Wight, England, UK. Earth Environ. Sci. R. Soc. Edinb..

[41] Linnaeus, C. Systema Naturae per Regna tria Naturae, secundum classes, ordines, genera, species, cum characteribus, differentiis, synonymis, locis. T. 1. *Holmiae: Impensis Direct. Laurentii Salvii.* 800 (1758).

[42] Greenwalt DE (2019). Diptera of the middle Eocene Kishenehn Formation. I. Documentation of diversity at the family level. Palaeontol. Electron..

[43] Hennig W (1973). Flügelgeäder und System der Dipteren unter Berücksichtigung der aus dem Mesozoikum beschriebenen Fossilien. Beiträge zur Entomologie.

[44] Osten Sacken CR (1865). Description of some new genera and species of North American Limnobina. Part I. Proc. Entomol. Soc. Phila..

[45] Schummel TE (1829). Beschreibung der in Schlesien einheimischen Arten einiger Dipteren-Gattungen 1 Limnobia. Meigen. Beitrage zur Entomologie, besonders in Bezug auf Schlesien, Breslau.

[46] Alexander CP (1922). New or little-known Tipulidae (Diptera) IX. Australasian species. Ann. Mag. Nat. Hist..

[47] Enderlein G (1938). 1912. Die Phoridenfauna Südbrasiliens. Stettiner Entomologische Zeitung.

[48] Brunetti E (1918). Revision of the Oriental Tipulidae with descriptions of new species. Part II. Rec. Indian Mus..

[49] Alexander CP (1928). Diptera. Fam. Tipulidae. Subfam. Cylindrotominae. Genera Insectorum..

[50] Meigen, J. W. Systematische Beschreibung der bekannten europäischen zweiflügeligen Insecten. I. *Friedrich Wilhelm Farstmann, Aachen*, i–xxxvi+1–332+[1] (1818).

[51] Kopeć K, Krzemiński W, Skowron K, Coram R (2017). The genera *Architipula* Handlirsch, 1906 and *Grimmenia* Krzemiński & Zessin, 1990 (Diptera: Limoniidae) from the Lower Jurassic of England. Palaeontol. Electron..

[52] Self JG, Johnson RC, Brownfield ME, Mercier TJ (2010). Stratigraphic cross sections of the Eocene Green River Formation in the Piceance Basin, northwestern Colorado: US. Geol. Surv. Digit. Data Ser. DDS-69-Y.

[53] Goloboff PA, Farris J, Nixon K (2008). TNT, a free program for phylogenetic analysis. Cladistics.

[54] Goloboff PA, Catalano S (2016). TNT version 1.5, including a full implementation of phylogenetic morphometrics. Cladistics.

[55] Geinitz FE (1884). Über die Fauna des Dobbertiner Lias. Zeitschrift der Deutschen Geologischen Gesellschaft.

[56] Handlirsch, A. Die fossilien Insecten und die Phylogenie der rezent Formen. Engelmann, Leipzig, (1906–1908).

[57] Maddison, W. P. & Maddison, D. R. Mesquite: a modular system for evolutionary analysis. Version 3.61. http://mescuiteproject.org. Accessed 22 June 2020 (2009).

[58] Nixon, K. C. WinClada ver. 1.00.08 Published by the author, *Ithaca, New York* (2002).

[59] Nixon, K. C. ASADO, version 1.85 TNT-MrBayes Slaver (vl 5.30). Published by the author, Ithaca, New York (2004).

