# Supplementary material for: Phylogeny and biogeography of the enigmatic ghost lineage Cylindrotomidae (Diptera, Nematocera)
Source: Sci Rep. 2021 Jul 6;11:13916. doi: 10.1038/s41598-021-91719-w (PMC8260593; doi:10.1038/s41598-021-91719-w)
Supplement: Supplementary file 1 — Supplementary Information. [file 41598_2021_91719_MOESM1_ESM.docx]

**Phylogeny and biogeography of the enigmatic ghost lineage Cylindrotomidae** **(Diptera, Nematocera)**

Iwona Kania-Kłosok^1*^, André Nel^2^, Jacek Szwedo^3^, Wiktoria Jordan-Stasiło^4^, Wiesław Krzemiński^5^

**Supplementary information**

**Supplementary Data S1**

Cylindrotomidae is represented by nine extant genera: *Cylindrotoma*, *Diogma*, *Liogma*, *Phalacrocera*, *Stibadocera*, *Stibadocerella*, *Stibadocerina*, *Stibadocerodes*^[49]^, and *Triogma*. The Stibadocerinae comprise the four genera *Stibadocerodes*, *Stibadocera*, *Stibadocerella*, and *Stibadocerina*. The Cylindrotominae contain the genera *Cylindrotoma*, *Cyttaromyia*, *Diogma*, *Liogma*, *Phalacrocera*, and *Triogma*.

**Supplementary Data S2**

***Cyttaromyia obdurescens*** Cockerell, 1925^[27]^

(Fig. 1)

**Redescription.** Head (Fig. 1A, C) small, not well-preserved, eyes well-developed, large, occiput dark brown colored. Thorax brown (Fig. 1A) only approximately 1.5x as long as head, pronotum dark-brown. Sc (Fig. 1B, E, F) terminating in C well beyond level of fork of Rs, opposite approximately half length of R_2+3+4_; crossvein sc-r approximately one of its length from tip of Sc; R_2+3+4_ only shorter than d-cell and d`-cell; R_3+4_ longer than d-cell and d`-cell; section of R_5_ from r'-m' connection to margin of wing shorter than d`-cell about 0.2x of length of this section; R_5_ evenly curved towards margin wing; crossvein r-m positioned d-cell slightly distad of fork of Rs; four medial branches of Mb present; d-cell elongate, gradually widening towards wing margin, d-cell longer than apical sections of M_1_ – M_4_; d-cell set noticeably more basad than supernumerary cell (d'-cell), approximately 3.5x as long as wide; d'-cell noticeably narrows basad, approximately 5.5x as long as wide; crossvein m-cu located beyond fork of Mb on M_1+2_ and M_3+4_; base of apical part of M_1_ almost perpendicular to M_2_, before turning towards apex of wing; M_1_ (excluding base of M_1_, before turning towards apex of wing) and M_2_ almost equal in length, slightly shorter than M_3_ and slightly longer than M_4_; apical section of M_3_ subsinuous from d-cell towards wing margin; distance between tip of R_5_ and tip of M_1_ or tip of M_1_ and tip of M_2_ almost equal in length; distance between tip of M_3_ and M_4_ 2x as long as distance between tip of M_2_ and M_3_; distances between tip of M_2_ and M_4_ and between tips of M_4_ and Cu almost equal in lengths. A_2_ elongate, slightly sinusoidal. Tip of A_1_ positioned behind fork of Rs. Haltera (Fig. 1D) elongate, shorter than 0.25x of fore wing.

Abdomen (Fig. 1G) approximately 5x as long as thorax, ovipositor not very elongate.

***Cyttaromyia gelhausi* sp. nov.**

http://zoobank.org/urn: lsid:zoobank.org:act:9165E3D2-514B-4524-9F85-A9895CBF2A31

(Figs 2 – 3)

**Description.** Body length 6.24 – 6.83 mm (male) (Fig. 2A), 6.72 mm (female) (Fig. 2D), wing length 7 mm (male) (Fig. 2A, 3A-C), 6.89 mm (female) (Fig. 2D). Head 1.10 – 1.93 mm wide (male) (Fig. 2A, B), 1.5 mm (female), small, not well-preserved, compound eyes well-developed, large; occiput dark brown colored; last segment of antennae rather elongate, slightly longer than penultimate one; antenna poorly preserved; palpi not visible.

Thorax brown (Fig. 2A) approximately 2.5x as long as head, pronotum dark-brown. Wing length 6.59 mm, width 1.48 mm (male), 6.35 – 7.47 mm, width 1.50 – 1.70 mm (female) with a pronounced stigma in distal part of wing; Sc (Fig. 3A-C) terminating in C just beyond level of fork of Rs, opposite approximately 1/10 length of R_2+3+4_; position of crossvein sc-r approximately one of its length from tip of Sc; R_2+3+4_ shorter than d-cell and d`-cell; R_3+4_ longer than d-cell and d`-cell; section of R_5_ from r'-m' connection to margin of wing shorter than d`-cell about 0.25x length of this section; R_5_ evenly curved towards margin wing; crossvein r-m situated slightly distad of fork of Rs; four medial branches of Mb presented; d-cell elongate, gradually widening towards wing margin, d-cell longer than apical sections of M_1_ – M_4_; d-cell set noticeably more basad than supernumerary cell (d'-cell), approximately 2.5x as long as wide; d'-cell noticeably narrows basad, approximately 3.5x as long as wide; crossvein m-cu located at fork of Mb on M_1+2_ and M_3+4_; base of apical part of M_1_ almost perpendicular to M_2_, before turning towards apex of wing; M_1_ (excluding base of M_1_, before turning towards apex of wing) and M_2_ almost equal in length, slightly shorter than M_3_ and slightly longer than M_4_; apical section of M_3_ subsinuous from d-cell towards wing margin; distance between tip of R_5_ and tip of M_1_ or tip of M_1_ and tip of M_2_ almost equal in length; distance between tip of M_3_ and M_4_ 2x as long as distance between tip of M_2_ and M_3_; distance between tip of M_2_ and M_4_ longer than distance between tips of M_4_ and Cu. Haltera elongate, shorter than 0.25x of wing.

Abdomen (Fig. 2A, C, D) approximately 2.5x as long as thorax, ovipositor not very elongate, 1.37 mm long, about 0.3x length of abdomen; hypopygium 1.09 mm long, wide, rather short, gonocoxites wide, massive.

***Cyttaromyia freiwaldi* sp. nov.**

(Figs 4 – 5)

http://zoobank.org/urn: lsid:zoobank.org:act:DB604450-AD47-4644-82E0-A33FEAEB7157

**Description.** Body (Figs 4B; 5A, B) length 4.8 mm, wing length 6.14 – 6.22 mm (Figs 4B, C, D; 5 A, B, E, F). Head (Figs 4A, B; 5C) 1.33 mm wide, compound eyes well-developed, large, occiput dark brown colored; antenna rather short, not very well preserved, 2.32 – 2.36 mm; palpi not well visible.

Thorax brown (Figs 4B; 5A, B) approximately 2.5x as long as head, pronotum brown. Wing length 6.14 – 6.22 mm, with a pronounced stigma in distal part of wing; Sc (Fig. 4C, D) terminating in C just beyond level of fork of Rs, opposite approximately 1/10 the length of R_2+3+4_; crossvein sc-r approximately one of its length from tip of Sc; R_2+3+4_ shorter than d-cell and d`-cell; R_3+4_ longer than d-cell and d`-cell; section of R_5_ from r'-m' connection to margin of wing shorter than d`-cell about 0.2x length of this section; R_5_ evenly curved towards margin wing; crossvein r-m positioned slightly distad of fork of Rs; four medial branches of Mb presented; d-cell elongate, gradually widening towards wing margin, d-cell longer than apical sections of M_1_ – M_4_; d-cell set noticeably more basad than supernumerary cel (d'-cell), approximately 3.5x as long as wide; d'-cell only slightly narrows basad, approximately 3.5x as long as wide; crossvein m-cu located beyond fork of Mb on M_1+2_ and M_3+4_ in 0.2 length of M_3+4_; base of apical part of M_1_ almost perpendicular to M_2_, before turning towards apex of wing; M_1_ (excluding base of M_1_, before turning towards apex of wing) and M_2_ almost equal in length, longer than M_3_ and M_4_; apical section of M_3_ almost straight from d-cell towards wing margin; distances between tip of R_5_ and tip of M_1_ or tip of M_1_ and tip of M_2_ almost equal in length; distance between tip of M_2_ and M_3_ 1.5x as long as distance between tip of M_3_ and M_4_; distance between tip of M_2_ and M_4_ longer than distance between tips of M_4_ and Cu. Haltera elongate, shorter than 0.2x of wing.

Abdomen (Figs 4B; 5A, B, D) approximately 5x as long as thorax, ovipositor not very long, 2.4 mm long, about 0.25x length of abdomen.

**Supplementary Data S3**

Characters scored for phylogenetic analysis.

1. Proportion between length of antenna and length of body: antennae one-third body length or shorter (0); antennae comparatively long, longer than one-third body length (1).

2. Shape of flagellomeres: not serrate (0); serrate (1).

3. Proportion between length and width of first and second flagellomeres: less than 3x longer than wide (0); more than 3x longer than wide (1).*

4. Coloration of wing: hyaline (0); at least partially pigmented brownish (1).

5. Length of vein Sc: Sc rather elongate, terminate opposite fork of Rs level or beyond (0); Sc short, terminate before fork of Rs level (1).

6. Tip of Sc: Sc reaching wing margin (0); Sc partially atrophied, not reaching wing margin (1); tip of Sc completely atrophied, not reaching wing margin (2).

7. Crossvein sc-r: well-developed (0); tendency to atrophy (1).

8. Branches of radial vein Rb: four branches merging into wing apex (0); three branches merging into wing apex (1); two branches merging into wing apex (2).

9. Number of branches of Rs reaching wing margin: at least two (0); one (1).

10. Length of vein R_1_: R_1_ terminating beyond level of fork of M_4_ and M_3_ (0); R_1_ terminating at level of fork of M_4_ and M_3_ (1).

11. Vein R_5_: well-developed (0); atrophied (1).

12. Shape of base of R_2+3+4_: slightly waved (0); strongly curved (1).

13. Position of R_5_: R_5_ separated from point of connection of r-m and M_1+2_ (0); R_5_ separated from margin of d-cell (1).

14. Relationship of M_1_ and M_2_: at least partially separate (0); fused (1).

15. Presence of petiole: absent (0); present (1).

16. A supernumerary crossvein r`-m` connected vein R_5_ with M_1_ near its origin, to produce two discal cells: absent (0); present (1).

17. R_3_ and R_4_: separate (0); fused (1).

18. Position of crossvein m-cu relative to bifurcation of M_3+4_: m-cu located at fork of M_3+4_ (0); m-cu located at extreme base of discal cell or before it (1).

19. Position of crossvein m-cu relative to bifurcation of Mb: beyond fork of Mb (0); at fork of Mb (1).

20. Shape of d-cell: rhomboid (0); rectangular (1).

21. Position of medial veins: M_1+2_ or M_2_ and M_3_ separated (0); M_1+2_ and M_3_ having at least a point of contact at base (1).

22. Shape of base of M_3_: not indented (0); strongly indented (1).

23. The distance between tip of M_4_ and tip of Cu: narrow (0); very wide (1).

24. Crossvein r-m: well-developed (0); reduced (1).

25. Position of A_2_ tip: beyond Rs origin level (0); before of Rs origin level (1).

26. Position of A_1_ tip: at or beyond of d-cell level (0); before of d-cell level (1).

27. Gonostylus: two-branched with well-developed clasper and lobe (0); two-branched, but with reduced lobe (1); with a single branch (2).*

28. Aedeagus: single (0); bifid (1); trifid (2).*

**Supplementary tables**

**Supplementary Table S1.** List of fossil species of *Cyttaromyia* (partially after used information from ^[26, 40]^).

| **species** | **age (ma)*** | **type horizon** |
| --- | --- | --- |
| *Cyttaromyia fenestrata* | 37.2 – 33.9 | USA/Green River Formation |
| *Cyttaromyia frelloi* | 38.0 – 33.9 | Baltic amber |
| *Cyttaromyia fuscula* | 37.2 – 33.9 | USA/Green River Formation |
| *Cyttaromtia lynnae* | 46.6 – 45.8 | USA/Kishenehn Formation |
| *Cyttaromyia obdurescens* | 50.3 – 46.2 | USA/Green River Formation/Roan Moutains/Colorado |
| *Cyttaromyia princetoniana* | 37.2 – 33.9 | USA/Florissant Formation |
| *Cyttaromyia quievreuxi* | 33.9 – 28.1 | France/Middle Salt Formation |
| *Cyttaromyia rayona* | 38.0 – 33.9 | Russia/Biamo Formation |
| *Cyttaromyia reclusa* | 50.3 – 46.2 | USA/Green River Formation/Roan Moutains/Colorado |
| *Cyttaromyia rossi* | 38.0 – 33.9 | UK/Isle of Wight/Bembridge Marls |
| *Cyttaromyia scudderi* | 56.0 – 47.8 | Denmark/Ølst Formation |
| *Cyttaromyia vahldieki* | 56.0 – 47.8 | Denmark/Ølst Formation |

* Ages are given after ^[6, 41]^

**Supplementary Table S2.** Matrix of characters

|  | **15** | **16** | **17** | **18** | **19** | **20** | **21** | **22** | **23** | **24** | **25** | **26** | **27** | **28** |
| --- | --- | --- | --- | --- | --- | --- | --- | --- | --- | --- | --- | --- | --- | --- |
| *Architipula*  *seebachi* | 1 | 0 | 0 | 0 | 0 | 0 | 0 | 0 | 0 | 0 | 1 | 1 | ? | ? |
| *Cylindrotoma*  *distinctissima* *distinctissima* | 0 | 0 | 1 | 1 | 0 | 1 | 0 | 0 | 0 | 0 | 1 | 0 | 2 | 2 |
| *Cyttaromyia*  *fenestrata* | 0 | 1 | 1 | 1 | 0 | 1 | 0 | 0 | 0 | 0 | 0 | 1 | ? | ? |
| *Diogma*  *glabrata* | 0 | 0 | 1 | 1 | 0 | 1 | 0 | 0 | 0 | 0 | 0 | 0 | 0 | 1 |
| *Liogma*  *nodicornis* | 0 | 0 | 1 | 1 | 1 | 1 | 0 | 0 | 0 | 1 | 0 | 1 | 2 | 2 |
| *Phalacrocera*  *replicata* | 0 | 0 | 1 | 1 | 1 | 1 | 1 | 0 | 0 | 0 | 0 | 0 | 2 | 2 |
| *Triogma*  *trisulcata* | 0 | 0 | 1 | 1 | 1 | 1 | 0 | 0 | 0 | 1 | 0 | 0 | 2 | 2 |
| *Tipula*  *oleracea* | 1 | 0 | 0 | 0 | 0 | 0 | 0 | 0 | 0 | 0 | 1 | 1 | 0 | 0 |
| *Stibadocera*  *bullans* | 0 | 0 | 2 | 1 | 0 | 1 | 0 | 1 | 1 | 0 | 0 | 0 | 1 | 2 |
| *Stibadocerella*  *pristina* | 0 | 0 | 2 | 1 | 0 | 1 | 0 | 1 | 1 | 0 | 0 | 1 | 0 | 2 |
| *Stibadocerina*  *chilensis* | 0 | 0 | 2 | 1 | 0 | 1 | 0 | 1 | 1 | 0 | 0 | 1 | 2 | 2 |
| *Stibadocerodes*  *australiensis* | 0 | 0 | 1 | 1 | 0 | 1 | 0 | 1 | 1 | 0 | 0 | 0 | 0 | 2 |

|  | **1** | **2** | **3** | **4** | **5** | **6** | **7** | **8** | **9** | **10** | **11** | **12** | **13** | **14** |
| --- | --- | --- | --- | --- | --- | --- | --- | --- | --- | --- | --- | --- | --- | --- |
| *Architipula*  *seebachi* | ? | ? | ? | 0 | 0 | 0 | 0 | 0 | 0 | 0 | 0 | 0 | 0 | 0 |
| *Cylindrotoma*  *distinctissima* *distinctissima* | 0 | 0 | 0 | 0 | 0 | 1 | 1 | 2 | 0 | 0 | 0 | 0 | 0 | 0 |
| *Cyttaromyia*  *fenestrata* | ? | 0 | ? | 0 | 0 | 0 | 0 | 2 | 0 | 0 | 0 | 0 | 0 | 0 |
| *Diogma*  *glabrata* | 0 | 0 | 0 | 0 | 0 | 2 | 0 | 2 | 0 | 0 | 0 | 1 | 0 | 1 |
| *Liogma*  *nodicornis* | 0 | 1 | 0 | 0 | 1 | 0 | 0 | 2 | 0 | 1 | 0 | 1 | 1 | 1 |
| *Phalacrocera*  *replicata* | 0 | 0 | 0 | 1 | 1 | 2 | 0 | 1 | 0 | 0 | 0 | 0 | 0 | 1 |
| *Triogma*  *trisulcata* | 0 | 1 | 0 | 0 | 0 | 1 | 1 | 1 | 0 | 1 | 0 | 1 | 1 | 1 |
| *Tipula*  *oleracea* | 0 | 0 | 1 | 1 | 1 | 2 | 0 | 0 | 0 | 0 | 0 | 0 | 0 | 0 |
| *Stibadocera*  *bullans* | 1 | 0 | 1 | 0 | 1 | 2 | 0 | 1 | 1 | 1 | 1 | 0 | 0 | 1 |
| *Stibadocerella*  *pristina* | 1 | 0 | 1 | 0 | 1 | 0 | 0 | 2 | 1 | 0 | 1 | 0 | 0 | 1 |
| *Stibadocerina*  *chilensis* | 1 | 0 | 1 | 0 | 1 | 0 | 0 | 2 | 1 | 0 | 1 | 0 | 0 | 1 |
| *Stibadocerodes*  *australiensis* | 1 | 0 | 1 | 0 | 1 | 0 | 0 | 2 | 0 | 0 | 1 | 0 | 0 | 1 |

**Supplementary figures**


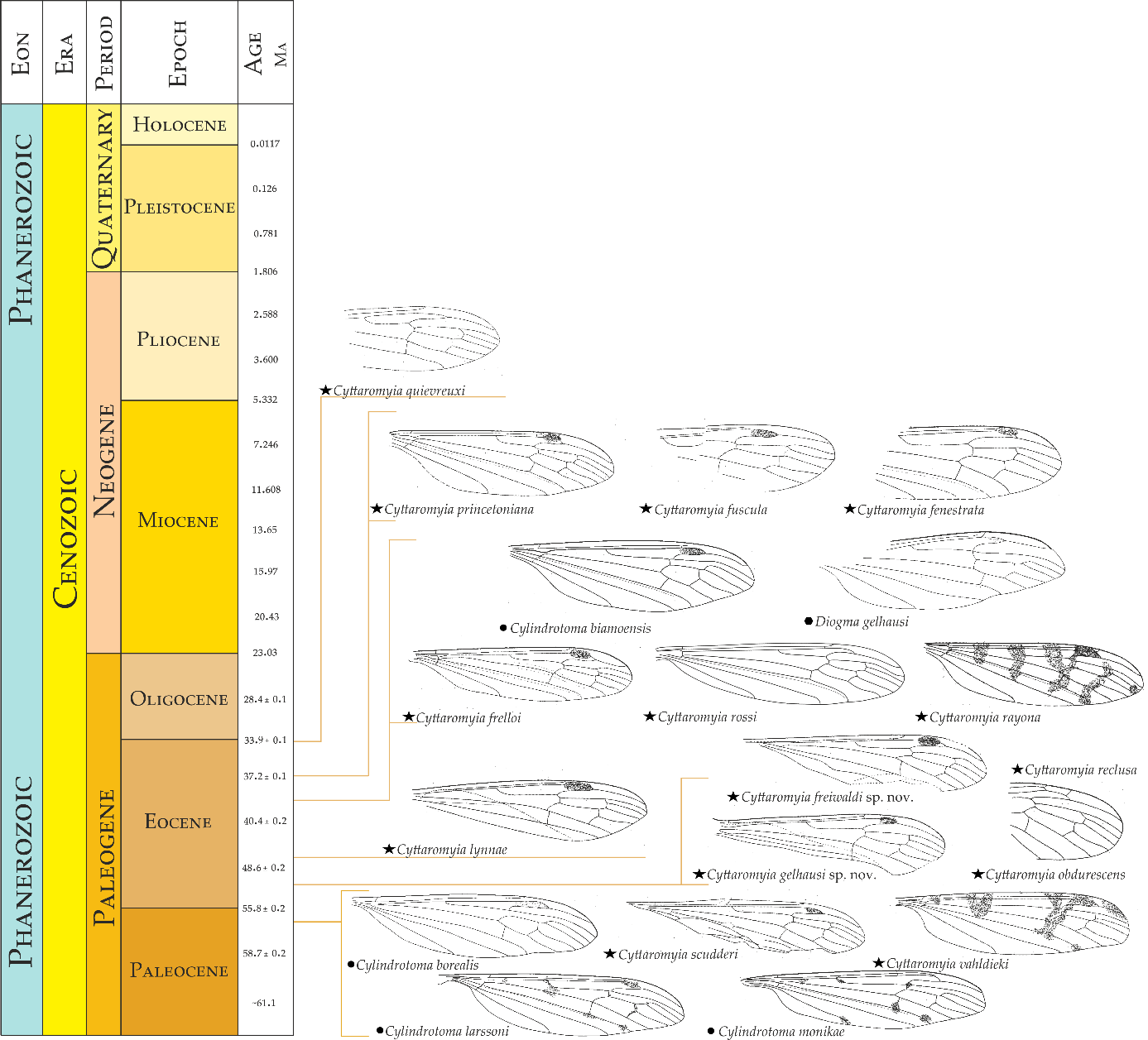


Supplementary Figure S1. Wing venation and color pattern of fossil Cylindrotominae with chronostratigraphic distribution view, wing venation redrawn^[24, 25, 27-29, 31, 34, 40, 48]^. Stratigraphic chart according to International Stratigraphic Chart, International Commission of Stratigraphy (v. 2021/05) https://stratigraphy.org/chart.


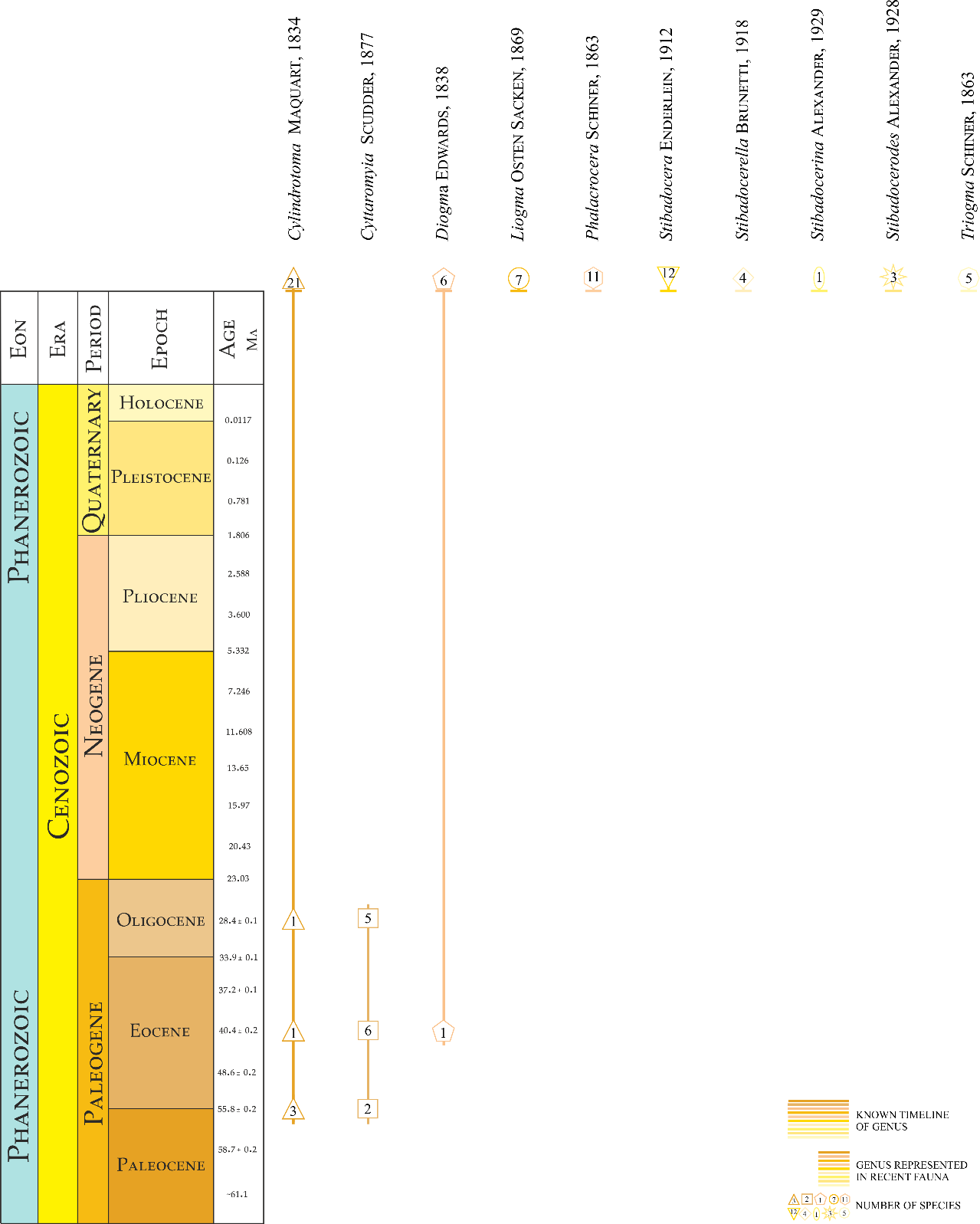


Supplementary Figure S2. Chronostratigraphic distribution of Cylindrotomidae. In white figures the number of species is given. Lines mark time of duration of the genus. Stratigraphic chart according to International Stratigraphic Chart, International Commission of Stratigraphy (v. 2021/05) https://stratigraphy.org/chart.


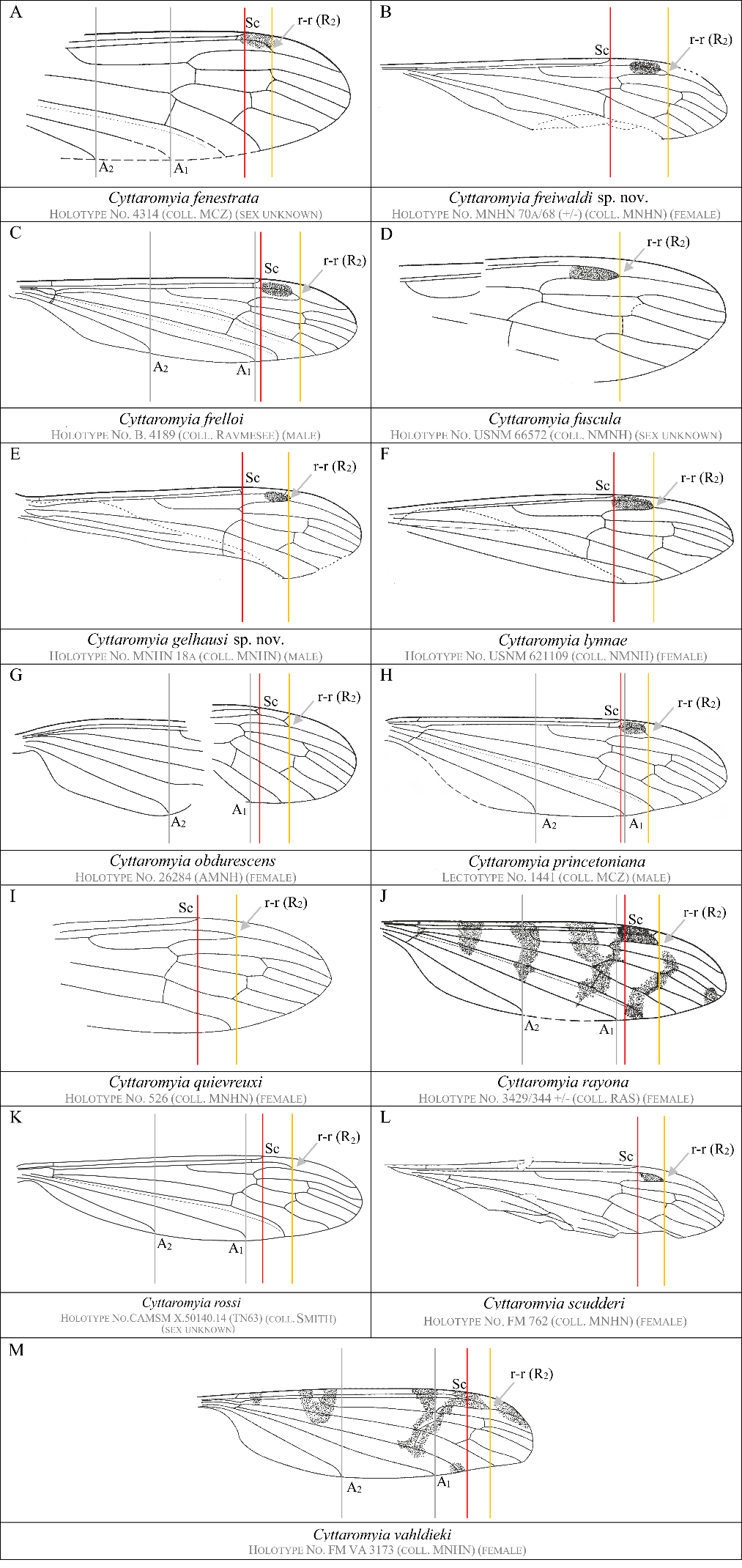


Supplementary Figure S3. Wing venation of species of *Cyttaromyia*, redrawn^[28-31, 39, 40]^. Abbreviations: lines cut the points: grey line – tips of A_1_ or A_2_; red line – tip of Sc; yellow line – to tip of r-r (R_2_).


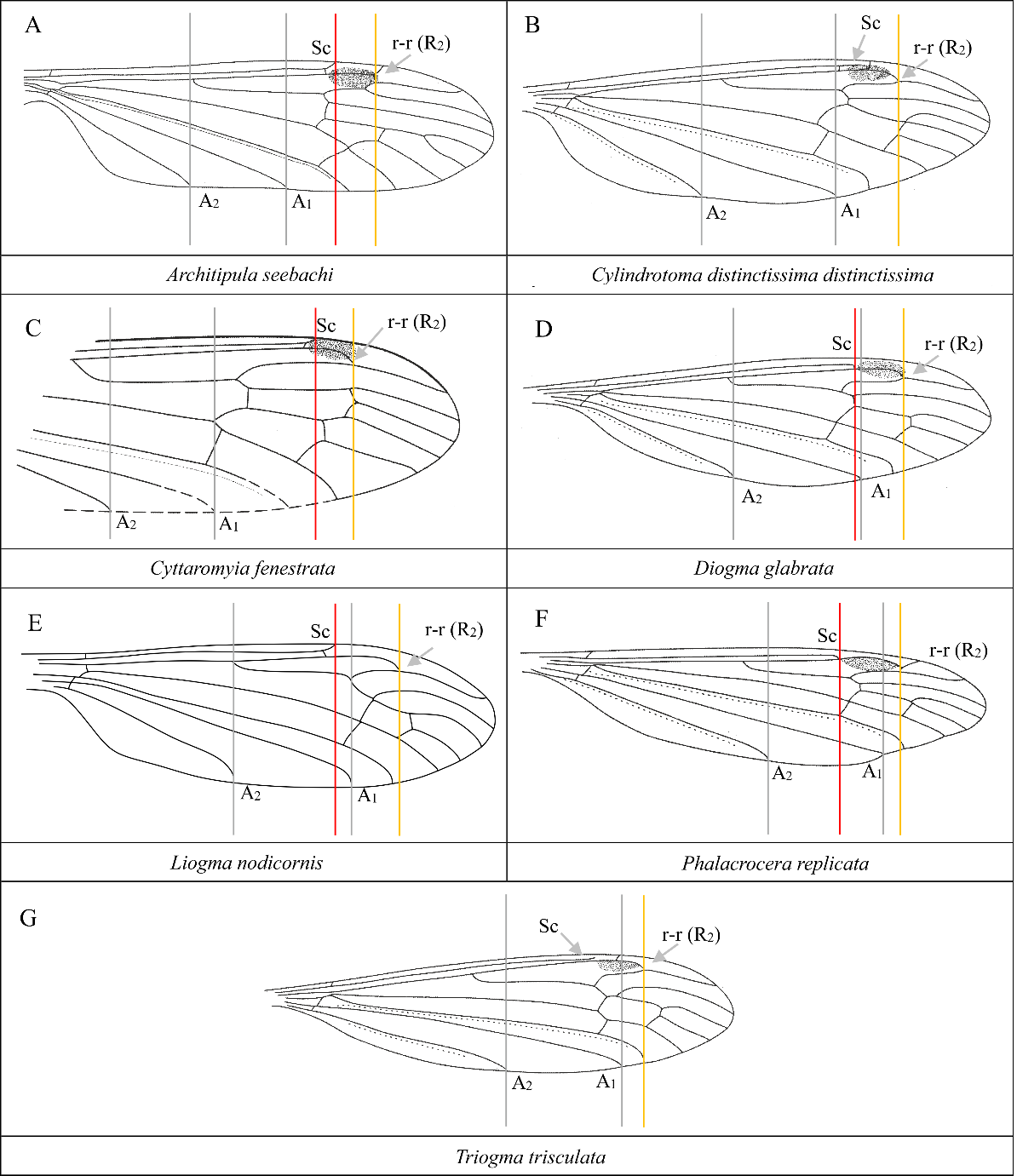


Supplementary Figure S4. Wing venation of type species of Cylindrotominae and the genus *Architipula* – as an outgroup used in parsimony analysis, redrawn^[39,49, 60]^. Abbreviations: lines cut the points: grey line – tips of A_1_ or A_2_; red line – tip of Sc; yellow line – to tip of r-r (R_2_).

**Supplementary references**

60. Alexander, C.P. Diptera of Patagonia and South Chile. Part I. Crane-flies (Tipulidae, Trichoceridae, Tanyderidae). *Diptera of Patagonia and South Chile*, **1**, 1-240 (1929).
